# Supplementary material for: Autophagy downregulation contributes to insulin resistance mediated injury in insulin receptor knockout podocytes in vitro
Source: PeerJ. 2016 Apr 11;4:e1888. doi: 10.7717/peerj.1888 (PMC4830256; doi:10.7717/peerj.1888)
Supplement: Data S1 [file peerj-04-1888-s001.docx]

|  |  |  |  |  |
| --- | --- | --- | --- | --- |
| **Figure 1A** |  |  |  |  |
| **Sample** | **Mean Relative Expression (IR)** | **SEM (+/-)** | **p value** |  |
| **CONTROL** | 0.7578 | 0.0358 | - |  |
| **IR shRNA** | 0,3501 | 0,0114 | 0,0004 |  |
|  |  |  |  |  |
| **Figure 1B** | **Mean Relative Expression (IR)** | **SEM (+/-)** | **p value** |  |
| **CONTROL** | 1 | 0 | - |  |
| **IR shRNA** | 0.1833 | 0.0088 | 0.0002 |  |
| **Figure 2A** | **Mean Relative Expression (BECLIN)** | **SEM (+/-)** | **p value** |  |
| **CONTROL** | 1.919 | 0.0423 | - |  |
| **IR shRNA** | 1.229 | 0.1056 | 0.0037 |  |
|  | **Mean Relative Expression (P62)** | **SEM (+/-)** | **p value** |  |
| **CONTROL** | 0.6004 | 0.0407 | - |  |
| **IR shRNA** | 0.8401 | 0.0494 | 0.0200 |  |
| **Figure 2B** | **Mean Relative Expression (LC3Ⅱ)** | **SEM (+/-)** | **p value** |  |
| **CONTROL** | 0.5447 | 0.0211 | - |  |
| **IR shRNA** | 0.3509 | 0.0355 | 0.0082 |  |
| **Figure 5A** | **Mean Relative Expression (NEPHRIN)** | **SEM (+/-)** | **p value** |  |
| **CONTROL** | 0.5264 | 0.0117 | - |  |
| **IR shRNA** | 0.4122 | 0.0174 | 0.0056 |  |
| **Figure 6A** | **Mean Relative Expression (NEPHRIN)** | **SEM (+/-)** | **p value** |  |
| **dmso** | 0.3730 | 0.0270 | - |  |
| **Rapa** | 0.7419 | 0.0132 | 0,0003 |  |
|  |  |  |  |  |
|  | **Mean Relative Expression P62** | **SEM (+/-)** | **p value** |  |
| **dmso** | 1.228 | 0.0365 | - |  |
| **Rapa** | 0.4451 | 0.0292 | P<0.0001 |  |
| **Figure6B** | **Mean Relative Expression (LC3Ⅱ)** | **SEM (+/-)** | **p value** |  |
| **dmso** | 0.3085 | 0.0163 | - |  |
| **Rapa** | 0.6357 | 0.0380 | 0.0014 |  |
